# Surgical navigation for targeted retroperitoneal lymph-node removal: a randomised, controlled, phase 3 trial

**DOI:** 10.1016/j.eclinm.2024.102754

**Published:** 2024-07-26

**Authors:** Harald C. Groen, Esther M.K. Wit, Wouter J. Heerink, Koert F.D. Kuhlmann, Jasper A. Nijkamp, Ruben van Veen, Ivo G. Schoots, Sara Balduzzi, Henry J.M.A.A. Zijlmans, Pim J. van Leeuwen, Henk G. van der Poel, Theo J.M. Ruers

**Affiliations:** a Department of Surgical Oncology, The Netherlands Cancer Institute, Amsterdam, the Netherlands; b Department of Urology, The Netherlands Cancer Institute, Amsterdam, the Netherlands; c Department of Radiology, The Netherlands Cancer Institute, Amsterdam, the Netherlands; d Department of Biometrics, The Netherlands Cancer Institute, Amsterdam, the Netherlands; e Department of Gynecology, The Netherlands Cancer Institute, Amsterdam, the Netherlands; f Department of Urology, Amsterdam University Medical Centers, Amsterdam, the Netherlands; g Nanobiophysics Group, Faculty TNW, University of Twente, Enschede, the Netherlands

**Keywords:** Surgical navigation, Retroperitoneal lymph node dissection, Electromagnetic tracking, 3D model for surgical localisation

## Abstract

**Background:**

Metastatic retroperitoneal lymph node dissection (LND) for nodal recurrence is applied for a variety of cancers, such as urological, gynaecological and rectal cancer. Precise localisation and resection of these lymph nodes (LNs) during surgery can be challenging, especially after previous radiotherapy or surgery. The objective of this study was to assess the added value of surgical navigation for targeted LND in the retroperitoneum.

**Methods:**

We performed an open-label randomised, controlled, phase 3 trial at the Netherlands Cancer Institute, Amsterdam. Eligible participants were over 18 years of age, scheduled for targeted retroperitoneal LND by laparotomy, with removal of one or more suspected (targeted) LN(s) as assessed by diagnostic imaging. Patients were randomised (1:1) between conventional LND and LND using surgical navigation, by means of a minimisation method stratified for tumour origin (urological, colorectal and other). For the surgical navigation, a digital 3D model of the patients' anatomy was created from diagnostic CT scans, including delineation of the targeted LN(s). The 3D model was linked to the patients’ position in the operation room. Using an electromagnetic tracking system, with a sterile tracked pointer, the actual position of the pointer was shown in the 3D model, enabling the surgeon to localize the targeted LN(s). The primary outcome of the study was the percentage of successful procedures. Success was defined as no residual target LN(s) visible on postoperative CT imaging. This study was registered with ClinicalTrials.gov, NCT05867095.

**Findings:**

From January 2017 to December 2020, 69 participants were included in the study, 35 (51%) in the conventional arm and 34 (49%) in the navigation arm. Four patients were not evaluable and excluded from further analysis; three in the conventional arm (patients withdraw from study participation), one in the navigation arm (discontinued surgery, misclassified diagnosis). According to intention-to-treat analysis, 50% (16/32) of the surgical procedures was successful in the conventional arm, versus 85% (28/33) in the surgical navigation arm (one-tailed p = 0.0028, 90% CI: 14%–56%). Using the Clavien-Dindo classification, the overall complication rate was comparable between the conventional arm and the navigation arm. Surgeons judged the surgical navigation setup as valuable, the median preference score to use surgical navigation was 3.7 (3.3–4.0) (scale 1–5), and the median system usability score was 75 (70–85) (scale 0–100).

**Interpretation:**

Surgical navigation allows for significantly better localisation and removal of target LN(s) in the retroperitoneum.

**Funding:**

This research was supported by the KWF-Alpe d'HuZes (NKI 2014-6596) and by an institutional grant of 10.13039/501100004622The Dutch Cancer Society and of the 10.13039/501100002999Dutch Ministry of Health, Welfare and Sport.

**Research in context**

**Evidence before this study**

Surgical removal of lymph nodes (LNs) in the retroperitoneum may be indicated, if tumour involvement is suspected on diagnostic imaging. In such cases, surgery can result in an improved disease-free survival. However, precise intra-operative localisation of these LN(s) can be challenging, especially after previous treatment. A possible solution to precisely localize targets within the body is surgical navigation. A technique that is well known for different applications like neurosurgery and orthopaedics. We conducted a PubMed search for articles published between 2010 and 2017, but unfortunately, we found no randomised clinical trials showing the additional benefit of this technique in localizing LNs in the retroperitoneum.

**Added value of this study**

The results of this randomised clinical trial show that surgical navigation improves the surgical outcome of targeted LN resections. Surgical navigation proved to be a valuable technique to locate targets within the retroperitoneum. This suggests that surgical navigation may be beneficial for high-precision surgeries in other fields as well. Key for the technology to be adapted by the surgeons was the intuitive setup which allows for seamless integration within the clinical workflow.

**Implications of all the available evidence**

Surgical navigation should be considered as a useful tool to optimize targeted LN dissections in the retroperitoneal area.

## Introduction

With the advancement of imaging techniques, small-millimetre scale tumour lesions are increasingly detected during follow up of cancer patients.^1^ Especially the more widespread use of MRI, and the application of target specific PET tracers has significantly improved cancer detection at such small volumes barely noticed in the past.^2^^,^^3^ Such patients with image detected recurrence of limited size represent a heterogeneous population with varying clinical outcome after targeted LND, depending on number and location of lesions as well as the time interval between primary tumour and recurrence. In colorectal and prostate cancer, patients with isolated retroperitoneal or pelvic nodal metastasis tend to have the most favourable outcome.^4^, ^5^, ^6^, ^7^ Although evidence for the best treatment option for these patients with oligometastatic cancer is missing, there is a tendency towards local directed therapy, either by stereotactic radiotherapy or LND.^5^^,^^8^, ^9^, ^10^ LNDs are often challenging due to previous surgery or local radiotherapy. As a result many patients show still persistent elevated tumour markers or PET positive tumour lesions at their first follow-up scan, varying from 25% to 63%, indicating that the suspected tumour lesions may be missed during surgery.^4^^,^^8^^,^^11^^,^^12^

A novel strategy to improve intra-operative tumour localisation during LND is surgical navigation. The technology is well established in neurosurgery, spine, knee and ENT surgeries^13^, ^14^, ^15^ and supported by several commercial suppliers (Medtronic, Brainlab, Stryker, and Karl Storz). It provides the surgeon with real time feedback on the position of the instruments in relation to the intra-operative anatomy. In earlier studies we proved the safety and accuracy of the technology for major pelvic surgery.^16^^,^^17^ In a retrospective cohort study it was demonstrated that surgical navigation significantly improved radical tumour resection (R0) of recurrent rectal cancer.^18^ There are no objective data, however, that surgical navigation could also be of value in localizing small tumour lesions, such as often present during lymph node recurrence.

Here, we perform a randomised controlled trial (RCT) to evaluate the efficacy of surgical navigation for retroperitoneal LN dissection. We hypothesized that surgical navigation during targeted LND is superior to the standard surgical procedure with regard to LN(s) localisation and resection.

## Methods

### Study design

This is an open-label randomised study conducted at a single centre, The Netherlands Cancer Institute. The study randomised between conventional LND and LND performed using surgical navigation. The trial was approved by the Medical Ethical Committee of The Netherlands Cancer Institute and the Dutch Central Committee on Research Involving Human Subjects (CCMO).

### Patients

Eligible patients were 18 years or older and diagnosed with one or more suspicious LN in the retroperitoneal space. On imaging, a LN was suspect when it was positive on PET-imaging (i.e. PSMA-PET or FDG-PET), and/or had a short-axis of ≥10 mm, and/or morphological characteristics (i.e. round, unilateral) on CT. All participants were planned for open surgical resection of at least one LN suspect on pre-operative imaging. Exclusion criteria consisted of any metal implants in the pelvic area and any contra-indication for intravenous CT contrast agent.

Patients were recruited by the medical team, and provided written informed consent before inclusion. Medical data required for this study was collected from the patient's medical record.

### Randomisation

Patients were randomly assigned (1:1) to either conventional LND (conventional arm) or LND using surgical navigation (navigation arm) by an investigator. Within the EDC system (ALEA), patients were randomised between the two arms using the minimization method. One stratification factor was used, namely the discipline of tumour origin (urological, colorectal, other). For each of the three categories, a threshold of 1 was used to balance the distribution between the randomisation results with a random element to ensure unpredictability. The scheme was blinded from the surgeons and investigators.

### Procedures

At least two weeks prior to surgery, a dual-phase contrast enhanced CT scan (arterial phase and delayed washout phase) was acquired. For the conventional arm, the suspected LN(s)–called the target LN(s)–were indicated on the CT scan. For the navigation arm, a patient-specific 3D model was created based on the dual-phase CT scan, consisting of the indicated target LN(s), bones, arteries, vessels and ureters. Prior to the surgery, the indicated target LN(s) were discussed and approved by the surgeon in both arms.

For the navigation arm, image-guided surgery was implemented as published previously.^16^, ^17^, ^18^ In short, prior to the start of the surgery, three electromagnetic (EM) patient trackers (Percunav, Philips Best, The Netherlands) were attached to the skin. Subsequently, a cone-beam CT (CBCT, Allura XperCT, Philips Best, The Netherlands) scan was acquired of the lower abdomen and pelvis under general anaesthesia with the patients in surgical position. A NDI Aurora TableTop Field Generator (Northern Digital Inc., Waterloo, Ontario, Canada) was placed underneath the patient, which allowed for simultaneously tracking of the patient trackers and a NDI EM tracked pointer for guidance during surgery. By registering the CBCT scan with the dual-phase pre-operative CT scan, the 3D model was matched with the patients' position on the surgical table and shown on a screen together with the EM pointer of the surgeon. The EM pointer enabled the surgeon to navigate through the patients’ anatomy and to locate the target LN(s).

For both arms, follow-up imaging, i.e. MRI or CT, was acquired within 3 months after surgery to verify removal of the targeted LN(s). Verification of LN removal was performed blinded and independently by two experienced researchers in medical imaging; if no consensus was reached, a radiologist was consulted for the final conclusion. If the results were still inconclusive, follow-up clinical and imaging data were used for verification of target removal.

In the navigation arm, post-operative questionnaires about the use of the surgical navigation were provided to the surgeons. Surgeons’ satisfaction of applying the technique during surgery was calculated on a Likert scale, ranging from 1 (preferred surgeries without navigation) to 5 (preferred surgeries with navigation). An average score higher than 3 is considered to be in favour of surgical navigation. In addition, the system usability score (SUS) was determined, ranging from 0–50 (no usable system), 50–70 (marginal usable system) to 70–100 (fully usable system).^19^

### Outcomes

The primary outcome of the study was the percentage of successful procedures, where success was defined as no remaining target LN(s) on follow-up imaging. Secondary outcomes were the overall surgical time, success rate of retrieved individual targeted LN(s), blood loss, surgeons' satisfaction of the procedure, complications and hospital stay. In addition, a comparative analysis was performed of LN size between LN(s) that were successfully removed and those that were left behind. Safety and adverse events were assessed during surgery and using the patient's clinical records during follow-up.

### Statistical analysis

The study was powered based on initial results of a pilot study.^16^ The success rate to remove all target LN(s) for the conventional arm was estimated to be 60% (pA = 0.60), while for the navigation arm it was estimated to be 84% (pB = 0.84). The null hypothesis H0: pA = pB was tested against the alternative hypothesis H1: pB > pA with one-sided type I error of 5% and power of 80%. With these hypotheses, the number of patients needed per arm was 41. Estimated accrual of the patients was 36 months.

The intention-to-treat (ITT) analysis was performed on all evaluable patients. The per-protocol (PP) analysis was performed on the patients that completed the full protocol, excluding clinical deviations, i.e. technical failures and non-resections of the target LN as decided by the surgeon during the intervention.

Continuous variables are reported as median (inter-quartile range (IQR)), and categorical variables as absolute numbers and percentages. Continuous variables are compared between the two arms with the Wilcoxon rank sum test, and categorical variables with the Fisher's Exact Test. A secondary analysis accounting for the difference in the number of LN(s) per patient was conducted: a generalized linear mixed model including the patients as a random effect was used. For the primary outcome, we report the difference in proportions of successful procedures between the two arms, along with the two-sided Wald confidence interval–with continuity correction at a 90% level–since its lower limit is equal to the lower limit of the one-sided 95% confidence interval. For the primary outcome the one-tailed Fisher test p-value is presented, while for other endpoints two-tailed p-values. A p-value of ≤0.05 is considered to be statistically significant. The statistical analyses were performed using R (4.3.0). There was no data monitoring committee installed for this study. The trial was registered as NCT05867095 at ClinicalTrials.gov.

### Role of the funding sources

The funders of the study had no role in study design, data collection, data analysis, data interpretation, writing of the report or decision to submit the paper for publication.

## Results

**Fig. 1:**
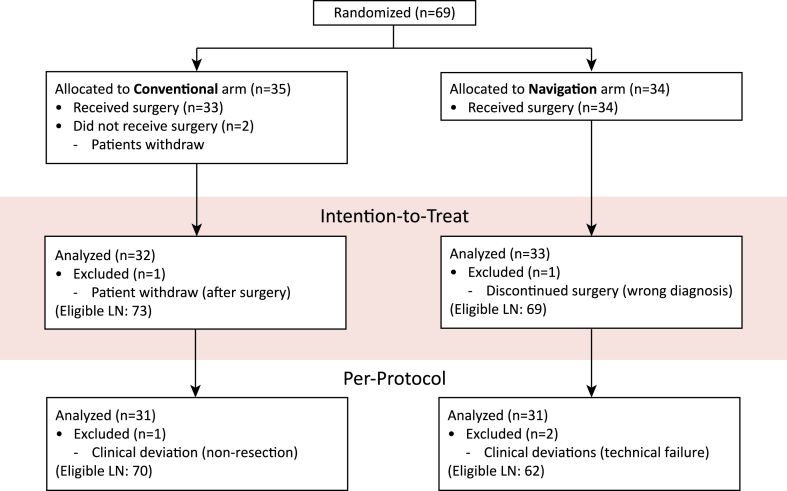
Consort flow diagram, with n = number of patients and LN = number of eligible target lymph nodes. Note that one patient can have one or more target LN(s). The intention-to-treat analysis includes all consented patients undergoing surgery for the correct diagnosis, the per-protocol analysis excluded clinical deviations, i.e. technical failures and non-resections of the target LN(s), as decided by intention during surgery.

The study started in January 2017. During the course of the study, surgeons clearly perceived the clinical benefits of the navigation procedure and became reluctant to further randomise patients. As such, the study was closed early in January 2021. At that time, 69 out of 82 patients were included, 35 (51%) in the conventional arm and 34 (49%) in the navigation arm (Fig. 1). Two patients in the conventional arm withdrew from the study, after reconsideration they did not want to undergo surgery as they believed that the LN(s) couldn't be removed without navigation. One patient in the conventional arm withdrew after surgery, but before the follow-up CT scan, without providing any reason. In the navigation arm, in one patient the surgical procedure was discontinued due to wrong diagnosis, i.e. the supposed targets were actually collateral vessels instead of LN(s). Leaving 32 (49%) evaluable patients in the conventional arm and 33 (51%) in the navigation arm. On post-operative imaging, consensus of LN removal between the researchers was 87.3% (124/142), in 12.7% (18/142) of the nodes a radiologist was consulted for final decision.

**Fig. 2:**
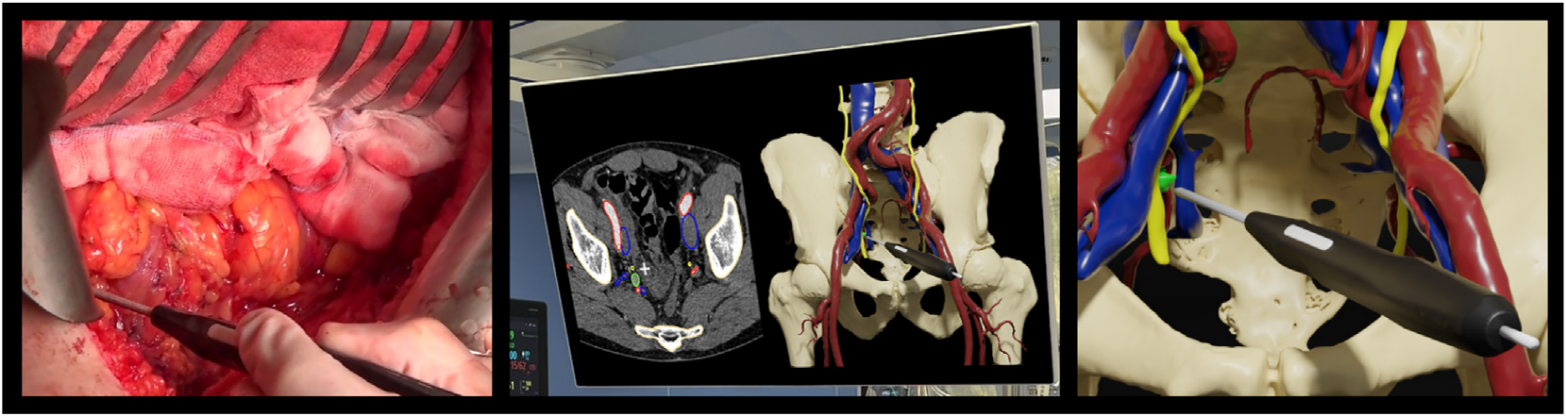
Schematic overview of live surgical navigation. Left: The surgical perspective during navigation, locating the target LN using the tracked pointer (black). Middle: OR screen displaying preoperative contrast enhanced CT scan with the location of the tracked pointer (white cross), and the patient's digital 3D model with the pointer (black). The 3D model is color-coded as follows; bone (yellow-white), urinary track (yellow), veins (blue), arteries (red) and target LN (green). Right: Close-up visualization of the 3D model and tracked pointer.

For the navigation arm, the patient's anatomy and LN(s) were visualised together with the EM pointer on a screen in the OR (Fig. 2). This navigation setup allowed the surgeon to navigate live through the patient's anatomy and locate the target LN(s) using the sterile EM pointer. Baseline characteristics were balanced between the two treatment arms, Table 1. In the navigation arm, there were more patients with a previous surgery (conventional arm 56%, 18/32 and the navigation arm 85%, 28/33).

**Table 1:** Baseline characteristics of the intention-to-treat population.

	Conventional arm (N = 32)	Navigation arm (N = 33)
Sex		
Female	13 (41%)	8 (24%)
Male	19 (59%)	25 (76%)
Age (years)	64.5 (56.5–72)	61 (54–68)
BMI (kg/m^2^)	26.8 (24.3–28.1)	26.5 (24.8–28.7)
Tumour group		
Urology	13 (41%)	20 (61%)
Colorectal	9 (28%)	7 (21%)
Other	10 (31%)	6 (18%)
Previous RTx		
No	14 (44%)	13 (39%)
Yes	18 (56%)	20 (61%)
Previous surgery		
No	14 (44%)	5 (15%)
Yes	18 (56%)	28 (85%)
Total number of target LN	73	69
Number of target LN per patient		
1	14	18
2	6	7
3	7	3
≥4	5	5
Size (cm)	1.5 (1.1–2.3)	1.3 (1.0–2.3)

Data are n, n (%), or median (IQR).

**Fig. 3:**
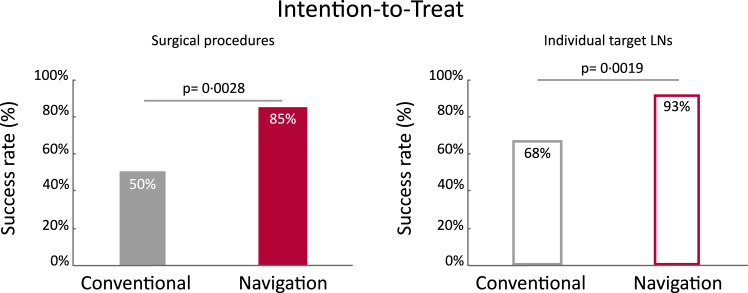
Left: Intention-to-treat analysis showing a significant difference (one-tailed p = 0.0028) in success between the conventional arm (50%, 16/32) and the navigation arm (85%, 28/33), in which success is defined per surgical procedure as removal of all target LN(s) in the patient. Right: The probability of successful LN removal of individual target LN(s) as predicted by the generalized linear mixed model, which was 68% for the conventional arm and 93% for the navigation arm (p = 0.0019).

For the ITT analysis, the number of successful surgeries, i.e. all target LN(s) were removed during surgery, was significantly higher (one-tailed p = 0.0028) in the navigation arm (85%, 28/33) compared to the conventional arm (50%, 16/32), Fig. 3. The difference between the two arms was 35% (90% CI: 14%–56%). At the LN level, the mixed model accounting for the correlation of LN(s) within the same patient, showed that the probability of successful LN removal was 93% for the navigation arm and 68% for the conventional arm (p = 0.0019). The total number of LNs removed per patient was not significant different (p = 0.24), i.e. for the conventional arm 9 (4.8–15) and for the navigation arm 5 (3–14). These combined findings indicate that navigation led to better targeted LN removal.

**Fig. 4:**
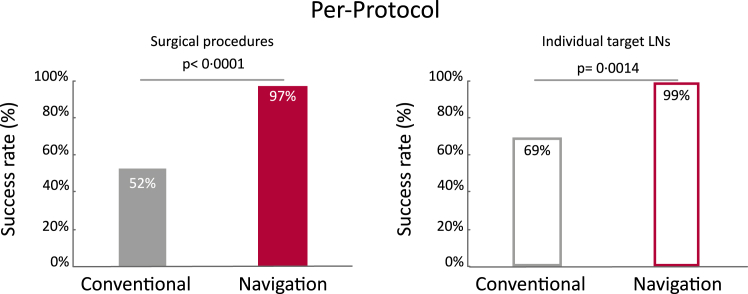
Per-protocol analysis excluding clinical deviations, i.e. technical failures and non-resections (n = 5) of the target lymph node (LN) as decided by intention during surgery. Left: Significant difference (one-tailed p < 0.0001) in success between of the conventional arm (52%, 16/31) and the navigation arm (97%, 30/31), in which success is defined per surgical procedure as removal of all target LN in the patient. Right: The probability of successful LN removal of the individual target LN(s) as predicted by the generalized linear mixed model, which was 69% for the conventional arm and 99% for the navigation arm (p = 0.0014).

For the PP analysis, the clinical deviations were excluded. In the conventional arm, this consisted of one patient in which resection of the target LNs was not performed due to unexpected presence of liver metastasis. In the navigation arm, navigation was not used in two patients due to technical failure (software) of the navigation setup. In addition, five target LN(s) that were identified, but deliberately not removed because the risk of complications, were excluded from the analysis as target LN. In this analysis, the number of successful surgeries in the navigation arm was significantly (one-tailed p < 0.0001) higher (97%, 30/31) compared to the conventional arm (52%, 16/31), Fig. 4. The difference between the two arms was 45% (90% CI: 30%–61%). At the LN level, the mixed model accounting for the correlation of LN(s) within the same patient, showed that the probability of successful LN removal was 99% for the navigation arm and 69% for the conventional arm (p = 0.0014).

**Fig. 5:**
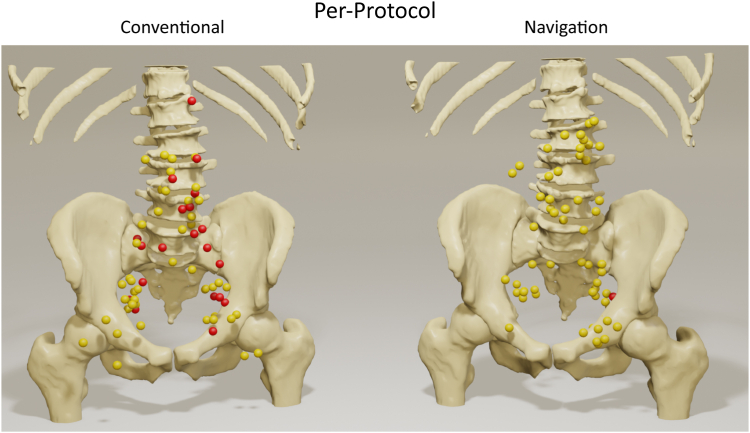
Compiled visualization of the per-protocol analysis of all successful removed LN (yellow) and not removed LN (red) in the conventional arm (left) and the navigation arm (right).

For the PP analysis, a compilation of the location of LNs of the conventional and the navigation arm are visualized in Fig. 5. A uniform distribution of the LNs within the body was observed, with no predominant regions where LN removal was not successful. For the conventional arm, the median size of the removed LN was 1.6 cm (1.2–2.4) and of the not removed LN was 1.2 cm (1.0–1.5). According to the mixed model accounting for the correlation of LN(s) within the same patient, there was no statistically significant impact of the LN diameter on the probability of a successful LN removal (p = 0.078).

### Secondary endpoints

The median time for setting-up the surgical navigation, i.e. sensor placement and CBCT acquisition, was 12 (12–16) minutes. The total surgery time was 2:38 (1:57–3:05) in the conventional arm, and 2:56 (1:56–4:16) in the navigation arm (p = 0.35). There were no complications specifically attributed to the use of surgical navigation. Using the Clavien-Dindo classification, the overall complication rate was comparable (p = 1) between the conventional arm and the navigation arm, at 30 days and 90 days after surgery, see Table 2. The median blood loss in the conventional arm was 200 (88–420) ml, in the navigation arm 250 (50–930) ml (p = 0.52). Median hospital stay after surgery was comparable between both arms (p = 0.84), 4 (3–7) days for the conventional arm and 4 (3–5) days for the navigation arm.

**Table 2:** Clavien-Dindo classification of surgical complications during, 30 days after and 90 days after the surgical interventions.

Complications	Conventional arm	Navigation arm	p-value
Number of patients	32	33	
Per-operative			
No	27 (84%)	27 (82%)	1
Yes	5 (16%)	6 (18%)	
30 days post-operative			
No	16 (50%)	18 (55%)	1
Yes			
I	3 (9%)	3 (9%)	
II	9 (28%)	6 (18%)	
III a	2 (6%)	4 (12%)	
III b	2 (6%)	2 (6%)	
90 days post-operative			
No	28 (88%)	28 (85%)	1
Yes			
II	2 (6%)	2 (6%)	
III a	0 (0%)	2 (6%)	
III b	1 (3%)	1 (3%)	
V	1 (3%)	0 (0%)	

Data are n, or n (%).

Per-operative complications consisted of either vessel, ureter, intestine and/or spleen damage.

**Fig. 6:**
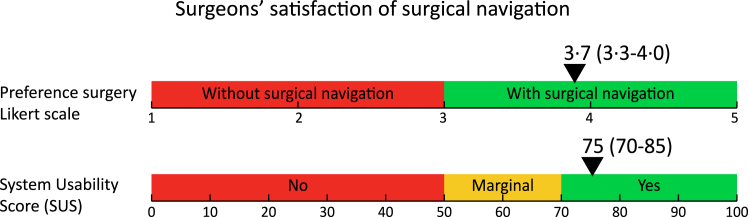
Surgeons' perspective on using surgical navigation (n = 30) expressed in a median (IQR) preference score to use the technique (Likert scale) and usability of the system (SUS).

In the navigation arm, surgeons completed 30 out the 31 questionnaires. Surgeon's satisfaction as measured by the post-surgery questionnaire was high; the median preference score to use surgical navigation was 3.7 (3.3–4.0) and the system usability score 75 (70–85), Fig. 6.

## Discussion

To our knowledge, this is the first randomised study showing benefit of surgical navigation during LND removal. In patients undergoing retroperitoneal LND, we have shown that surgical navigation resulted in superior (85%, 28/33) target localisation procedures of small tumour deposits compared to conventional surgery (50%, 16/32). The procedure proved safe and accurate, and surgeons judged the navigation procedure as helpful according to the Likert score and SUS score.

The results of our conventional arm on surgery success showed similar success rates (50%) compared to literature, ranging from 37% to 75%.^4^^,^^8^^,^^11^^,^^12^ As such, the success rate of the navigation arm (85%) outperformed the conventional arm and the success rates of conventional surgery, as reported in literature.

Surgical navigation in the retroperitoneum or pelvic area has hardly been described in literature.^16^, ^17^, ^18^^,^^20^, ^21^, ^22^, ^23^, ^24^ Two groups investigated the feasibility of navigation in small pilot studies for Transanal Minimal Invasive Surgery–Total Mesorectal Excision (TAMIS-TME).^20^, ^21^, ^22^, ^23^ Both groups concluded that navigation was feasible and safe, with satisfactory accuracy for clinical use. In earlier work we investigated the efficacy of surgical navigation for more extensive rectal surgery. In a retrospective case controlled cohort study, we observed a R0 resection for recurrent rectal cancer of 78.9% in the surgical navigation group versus 48.8% the standard surgery group.^18^ Recently, the utility of surgical navigation for advanced rectal cancers was confirmed by a prospective study of Solbakken et al.^24^ Both studies concentrated on the use of surgical navigation in clearly marked tumour volumes, guiding the surgeon to the right resection plane. In the current study we focused, however, on the utility of navigation for target localisation of small tumour lesions instead of determining the most accurate resection plane.

The present study shows that real-time intraoperative guidance is valuable during oncological surgery to perform successful targeted LN resections. Two other promising real-time intraoperative technologies are based on radioguidance and fluorescence. Radioguided surgery during open and robot-assisted surgery can help to identify cancerous LN(s) that might otherwise be missed.^5^^,^^25^ Fluorescence imaging in the near-infrared spectrum has shown potential for guiding surgeons during complex interventions.^26^ As such, surgical navigation and radio/fluorescence-guided surgery can complement each other. Surgical navigation can provide the most optimal route towards the target location, while fluorescent guided surgery and radio-guided surgery aim at indicating the precise location once the lesion is approached. As a result, combing these real-time intraoperative techniques might be useful in the future.

The primary end-point of this study was to evaluate the benefit of surgical navigation during selective LND removal. However, this study was not powered to assess the clinical benefit of selective LND removal. Larger multi-centre patient studies are required to assess the clinical value of this procedure.

We did not observe any complications attributed to the navigation technology. Thirty day complication rate was comparable between both arms, 12% gr III in the conventional arm versus 18% in the navigation arm, which is in concordance with literature on LND in colorectal cancer^8^ and prostate cancer.^11^

The median size of the suspected LN(s) in our study was 1.5 (1.1–2.3) cm in the conventional arm and 1.3 (1.0–2.3) cm in the navigation arm. We observed that for the conventional arm, the median size of the removed LN was 1.6 cm (1.2–2.4) and of the not removed LN was 1.2 cm (1.0–1.5), which is in concordance with the assumption that resections for small lesions might most benefit from navigation. Our study, however, has not been powered to do a formal sub-analysis on the size of the removed LN.

Baseline characteristics of both arms were comparable, with the exception of a higher number of patients in the navigation arm that underwent previous surgery. As previous surgery can result in a more challenging LND, it might be expected that this potential confounder could have negatively affected the success rate in the navigation arm. Despite, ITT analysis showed a significantly higher success rate in the navigation arm compared to the conventional arm. We observed that in the navigation arm five LN(s) were deliberately not resected because resection was considered unsafe. In the PP analysis these LN(s) were excluded, explaining the higher success rate in the navigation arm in the PP analysis.

In two patients (6%) we encountered a technical (software) failure. We expect that such failures will disappear with further development of the navigation setup for more widespread use.

One potential limitation is the assumption in the analysis that all surgeons would achieve equal benefit from using the navigation technique. However, given the relative low sample size and high number of different surgeons (25 in total, 19 different in the conventional arm, 17 different in the navigation arm), we could not adjust the results by fitting a mixed model with random effects for surgeon and surgeon by treatment.

In the present study intraoperative imaging was performed by CBCT. This technique is logistically tedious and difficult to repeat during the surgical procedure, when necessary. Intraoperative ultrasound, by means of an EM tracked ultrasound probe, seems far more attractive for intraoperative imaging as it can be performed in every operation theatre, is low cost and can be repeated when needed. We are currently investigating this approach.^27^

When we started our research, most LND were performed during open surgery. Nowadays, there is a strong shift towards minimal invasive robotic surgery for LND.^28^^,^^29^ The EM surgical navigation setup used in our study is compatible with robotic surgery, and not significantly affected by the metal of the surgical robot.^30^ Presently, a feasibility study is running on the use of surgical navigation for robot assisted LND.

In conclusion, in this randomised controlled trial we showed that surgical navigation improved complete LN removal in patients undergoing an open retroperitoneal targeted LND. The current study warrants further use of surgical navigation, especially in those surgeries where target lesions, such as small tumour positive LN(s), may be difficult to identify.

## Contributors

Conceptualization: KK; JN; HP; TR.

Data curation: HG; EW; WH; JN; RV; IS.

Data access: all authors.

Data verification: EW; WH; SB; TR.

Formal analysis: HG; RV; IS; SB; TR.

Funding acquisition: JN; TR.

Investigation: HG; EW; WH; KK; JN; RV; SB; HZ; PL; HP; TR.

Methodology: HG; KK; JN; RV; SB; TR.

Project administration: HG; WH; KK; JN; RV; TR.

Resources: HG; JN; RV; TR.

Software: HG; WH; JN; RV.

Supervision: KK; TR.

Validation: HG; EW; WH; RV; IS; SB; TR.

Visualization: HG; WH; TR.

Writing—original draft: HG; EW; WH; PL; HP; TR.

Writing—review & editing: HG; EW; WH; KK; JN; RV; IS; SB; HZ; PL; HP; TR.

## Data sharing statement

Non-public data.

## Declaration of interests

TR is involved as CMO of a company in surgical navigation, Bcon Medical.

## Appendix A Supplementary data

C1_Onderzoeksprotocol_V3.1_amendement
