# Supplementary material for: Surgical navigation for targeted retroperitoneal lymph-node removal: a randomised, controlled, phase 3 trial
Source: eClinicalMedicine. 2024 Jul 26;74:102754. doi: 10.1016/j.eclinm.2024.102754 (PMC11683954; doi:10.1016/j.eclinm.2024.102754)
Supplement: C1_Onderzoeksprotocol_V3.1_amendement [file mmc1.pdf]

Targeted Abdominal Lymph nodE dissections randomized for surgical NavigaTion

(TALENT)

Protocol title: "Targeted abdominal lymph node dissections randomized for surgical navigation"

**Protocol ID**	**NL58037.031.16/N16LND**
**Short title**	**Navigated abdominal lymph node dissection**
**EudraCT number**	
**Version**	**3.1**
**Date**	**10-1, 2017**
**Principal investigator**	*Prof. Th. J. M. Ruers, surgical oncologist*<br>*Division Surgical Oncology*<br>*NKI-AvL*<br>*Plesmanlaan 121, 1066 CX Amsterdam*<br>*T:+31 (0) 20 512 2538*<br>***t.ruers@nki.nl***
**Co-investigators**	*Dr. K.F.D. Kuhlmann, surgical oncologist*<br>*Division Surgical Oncology*<br>*NKI-AvL*<br>*Plesmanlaan 121, 1066 CX Amsterdam*<br>*T:+31 (0) 20 512 2344*<br>***k.kuhlmann@nki.nl***<br><br>*Prof. S. Horenblas, Urologist*<br>*Division of Urology*<br>*NKI-AvL*<br>*Plesmanlaan 121, 1066 CX Amsterdam*<br>*T:+31 (0) 20 512 2559*<br>***s.horenblas@nki.nl***<br><br>*Dr. H.G. van der Poel, Urologist*<br>*Division of Urology*<br>*NKI-AvL*<br>*Plesmanlaan 121, 1066 CX Amsterdam*<br>*T:+31 (0) 20 512 2556*<br>***h.vd.poel@nki.nl***

	Dr. J. Nijkamp, Postdoc<br>*Division Surgical Oncology*<br>*NKI-AvL*<br>*Plesmanlaan 121, 1066 CX Amsterdam*<br>*T:+31 (0) 20 512 1774*<br>***j.nijkamp@nki.nl***<br><br>*Dr. B. Pouw, Postdoc*<br>*Division Surgical Oncology*<br>*NKI-AvL*<br>*Plesmanlaan 121, 1066 CX Amsterdam*<br>*T:+31 (0) 20 512 1004*<br>***b.pouw@nki.nl***
**Sponsor**	***NKI-AVL***
**Subsidising party**	**n.a.**
**Independent expert**	*Dr. M.E. van Leerdam*<br>*Department of Gastroenterology and Hepatology*<br>*NKI-AVL*<br>*Plesmanlaan 121, 1066 CX Amsterdam*<br>*T:+31 (0) 20 512 1589*<br>***m.v.leerdam@nki.nl***

PROTOCOL SIGNATURE SHEET

**Name**	**Signature**	**Date**
**Vice-head of Department:**<br>*Prof. E. J. Th. Rutgers, Surgical oncologist*<br>*Vice-head of the Division Surgical Oncology*		
**Principal Investigator:**<br>*Prof. Th. J. M. Ruers, Surgical oncologist*<br>*Head of the Division Surgical Oncology*		

TABLE OF CONTENTS

1. INTRODUCTION AND RATIONALE ... 8
2. OBJECTIVES ... 10
3. STUDY DESIGN ... 10
4. STUDY POPULATION ... 12
4.1 Population (base) ... 12
4.2 Inclusion criteria ... 12
4.3 Exclusion criteria ... 12
4.4 Sample size calculation ... 12
5. TREATMENT OF SUBJECTS ... 13
5.1 Investigational treatment ... 14
5.2 Use of co-intervention (if applicable) ... 14
5.3 Escape medication (if applicable) ... 14
6. INVESTIGATIONAL PRODUCT ... 15
6.1 Name and description of investigational product(s) ... 15
6.2 Summary of findings from non-clinical studies ... 16
6.3 Summary of findings from clinical studies ... 16
6.4 Summary of known and potential risks and benefits ... 17
6.5 Description and justification of route of administration and dosage ... 17
6.6 Dosages, dosage modifications and method of administration ... 17
6.7 Preparation and labelling of Investigational Medicinal Product ... 17
6.8 Drug accountability ... 17
7. NON-INVESTIGATIONAL PRODUCT ... 18
8. METHODS ... 18
8.1 Study parameters/endpoints ... 18
8.1.1 Main study parameter/endpoint ... 18
8.1.2 Secondary study parameters/endpoints ... 18
8.1.3 Other study parameters (if applicable) ... 18
8.2 Randomisation, blinding and treatment allocation ... 19
8.3 Study procedures ... 19
8.4 Withdrawal of individual subjects ... 21
8.4.1 Specific criteria for withdrawal (if applicable) ... 21
8.5 Replacement of individual subjects after withdrawal ... 21
8.6 Follow-up of subjects withdrawn from treatment ... 21
8.7 Premature termination of the study ... 21
9. SAFETY REPORTING ... 22
9.1 Temporary halt for reasons of subject safety ... 22
9.2 AEs, SAEs and SUSARs ... 22
9.2.1 Adverse events (AEs) ... 22
9.2.2 Serious adverse events (SAEs) ... 22
9.2.3 Suspected unexpected serious adverse reactions (SUSARs) ... 23
9.3 Annual safety report ... 23

9.4 Follow-up of adverse events 23
9.5 Data Safety Monitoring Board (DSMB) / Safety Committee 23
10. STATISTICAL ANALYSIS 24
10.1 Primary study parameter(s) 24
10.2 Secondary study parameter(s) 24
10.3 Other study parameters 24
10.4 Interim analysis 24
11. ETHICAL CONSIDERATIONS 24
11.1 Regulation statement 24
11.2 Recruitment and consent 25
11.3 Objection by minors or incapacitated subjects (if applicable) 25
11.4 Benefits and risks assessment, group relatedness 25
11.5 Compensation for injury 25
11.6 Incentives (if applicable) 26
12. ADMINISTRATIVE ASPECTS, MONITORING AND PUBLICATION 27
12.1 Handling and storage of data and documents 27
12.2 Monitoring and Quality Assurance 27
12.3 Amendments 27
12.4 Annual progress report 27
12.5 Temporary halt and (prematurely) end of study report 27
12.6 Public disclosure and publication policy 28
13. STRUCTURED RISK ANALYSIS 28
14. REFERENCES 29

LIST OF ABBREVIATIONS AND RELEVANT DEFINITIONS

**ABR**	**ABR form, General Assessment and Registration form, is the application form that is required for submission to the accredited Ethics Committee (In Dutch, ABR = Algemene Beoordeling en Registratie)**
**AE**	**Adverse Event**
**CCMO**	**Central Committee on Research Involving Human Subjects; in Dutch: Centrale Commissie Mensgebonden Onderzoek**
**CT**	**Computed tomography**
**CV**	**Curriculum Vitae**
**DSMB**	**Data Safety Monitoring Board**
**EQ-5D-5L**	**Standardized questionnaire on outcome of health to be used for health technology assessment**
**EU**	**European Union**

**EudraCT**	**European drug regulatory affairs Clinical Trials**
**IC**	**Informed Consent**
**IMP**	**Investigational Medicinal Product**
**IMDD**	**Investigational Medicinal Device Dossier**
**LN**	**Lymph node**
**LND**	**Lymph node dissection**
**METC**	**Medical research ethics committee (MREC); in Dutch: medisch ethische toetsing commissie (METC)**
**MRI**	**Magnetic resonance imaging**
**N13NAV**	**Ongoing pilot study on the use of surgical navigation in the pelvic area**
**NKI-AvL**	**Netherlands cancer institute - Antoni van Leeuwenhoek**
**OR**	**Operating room**
**PET**	**Positron emission tomography**
**QLQ-C30**	**Standardized questionnaire on quality of life of cancer patients in general**
**QLQ-CR29**	**Standardized questionnaire on quality of life specifically for colorectal cancer patients**
**QLQ-PR25**	**Standardized questionnaire on quality of life specifically for prostate cancer patients**
**(S)AE**	**(Serious) Adverse Event**
**Sponsor**	**The sponsor is the party that commissions the organisation or performance of the research, for example a pharmaceutical company, academic hospital, scientific organisation or investigator. A party that provides funding for a study but does not commission it is not regarded as the sponsor, but referred to as a subsidising party.**
**SUSAR**	**Suspected Unexpected Serious Adverse Reaction**
**TME**	**Total mesorectal excision**
**Wbp**	**Personal Data Protection Act (in Dutch: Wet Bescherming Persoonsgevens)**
**WMO**	**Medical Research Involving Human Subjects Act (in Dutch: Wet Medisch-wetenschappelijk Onderzoek met Mensen**

SUMMARY

Rationale: Lymph node metastasis of pelvic tumours are primarily found along the iliac vessels, the caval vene, aorta, obturator regions, and in the mesorectal and pre-sacral space. Surgical removal of these lymph nodes may be indicated, especially if abnormal on pre-operative imaging. Exact localization of lymph nodes during surgery, however, can be challenging, especially after previous treatment (chemo-radiotherapy and/or previous surgery). We have developed a surgical navigation system which can aid in the localization and removal of lymph nodes. Within the system, the pre-operative imaging information is linked to the actual anatomy during surgery. The navigation system has been evaluated in a pilot study. In 25 patients, 67 of the 71 targeted lymph nodes on pre-operative imaging were actually localized during surgery and removed. These promising results have led to the design of a prospective randomized controlled trial in which the beneficial value of the navigation system for abdominal lymph node dissections will be evaluated.

Objective: To assess the beneficial value of our electromagnetic navigation system for targeted lymph node dissection in the abdomen.

Study design: Randomized controlled clinical trial.

Study population: All patients aged 18 or older, scheduled for open abdominal surgery in which targeted removal of one or more pathological lymph nodes is part of the surgical plan.

Intervention (if applicable): After providing informed consent, patients will be randomized between conventional surgery and navigation assisted surgery. In the control arm patients will be operated according to current clinical practice. In the experimental arm, patients will be operated with assistance of a navigation system.

Main study parameters/endpoints: Primary endpoint will be the number of failed procedures, in which failure is defined as: presence of any residual target lymph node on follow-up imaging. Secondary endpoints are: time from having the surgical field prepared for lymph node localization to actual removal of the lymph node, overall surgical time, individual LN retrieval rate, blood loss, operator satisfaction, complications, cost, hospital stay, and health related quality of life.

We estimate 40% failed procedures in the conventional arm, vs. 16% in the navigation arm. To achieve sufficient scientific power, we will need 41 patients in each arm.

Nature and extent of the burden and risks associated with participation, benefit and group relatedness: Patients will be asked to take quality of live and side effects questionnaires before surgery and at 3, 6 and 12 months after surgery. All patients will undergo one extra baseline contrast enhanced CT scan in the two weeks before surgery. For patients in the experimental arm, the CT scan is used to generate a 3D model of the anatomy. In the experimental arm, one intra-operative CT will be acquired to calibrate the navigation system. In both arms, the pre-operative CT might result in one

additional visit to our hospital. The surgeon is responsible for interpretation of the navigation system, and translation to surgical actions, therefore no additional risks are associated with participation.

1. INTRODUCTION AND RATIONALE

Clinical challenge:

Lymph node metastasis of pelvic tumours are primarily found along the iliac vessels, the caval vene, aorta, obturator regions, and in the mesorectal and pre-sacral space. In surgery of pelvic tumours, lymph node spread is taken into account by removal of more than just the primary tumour. For example, standard surgery for rectal tumours is done by performing a total mesorectal excision (TME), in which the entire mesorectum is removed, including the mesorectal and presacral lymph nodes [1]. For intermediate and high risk prostate cancer the surgical procedure consists of a prostatectomy plus a lymph node dissection (LND) along the internal and external iliac artery and the obturator region [2]. If pathologic lymph nodes outside the standard resection regions (extra-regional LN) are present, removal of these lymph nodes can be more challenging [3], [4], especially after previous treatments (chemo-radiotherapy and/or surgery).

Actual assessment of the presence of extra-regional LN is done with CT, PET, and/or MR. All the pre-operative information gathered from these scans is currently used during surgery by means of the “surgeon’s brain”, i.e. the surgeon reviews the information preoperatively and translates it into actions during surgery. Image guided navigation systems can be used to integrate preoperative imaging in the surgical procedure in such a way that pre-operative images are fitted in a 3D-environment available during surgery. The real-time positions of surgical tools can be related to the preoperative images during surgery, thus providing better assessment of resection planes and avoidance of vital structures.

Background of surgical navigation

In navigation surgery, pre-operative acquired images (e.g. CT, MRI, PET) are translated into patient specific 3D road maps. In order to utilize the road map, a tracking system needs to be used which links the roadmap to the actual anatomy of the patient on the operation table. Navigation during surgery results in 3D anatomical insight and offers a view beyond the surfaces of surgical planes and organs. This novel technique will be part of the operating room of the future and can lead to safer, tissue sparing and oncological more accurate procedures with an improved clinical outcome.

The application of image guided navigation is under investigation in many surgical fields. It is well established in surgical procedures of rigid structures such as bone and brain. Historically, neurosurgeons are the key players in this field and have already advanced to clinical studies to assess

the effect of their navigation techniques [5]. Navigation in neurosurgery originated from frame-based stereotactic procedures and is nowadays generally applied in the treatment of gliomas and brain metastases [5], [6]. In orthopaedic surgery navigation is used in pelvic screw fixation, leading to reduced malposition rates, and in musculoskeletal tumour resection [7]–[10]. ENT (ear, nose and throat) and maxillofacial surgeons apply navigation techniques in various procedures such as posttraumatic craniofacial reconstruction of the skull and cochlear implantation [11], [12].

The above-mentioned fields of surgery have in common that they are based on rather rigid, bone related, targets. Application of surgical navigation in the abdomen is more challenging, since organ movement, breathing motion and tissue deformation result in differences between the anatomical map and the actual anatomy. Only limited efforts have been taken to progress surgical navigation into this field. For example, Atallah et al. have reported on the use of surgical navigation in transanal TME, in three patients [13]. In their navigation setup, they assume that the anatomy is rigid, and cannot take anatomical changes into account.

During the last few years we have developed an innovative electromagnetic navigation system that may comply with the challenges for intra-abdominal navigation. In our setup, reference sensors are placed on the patient surface just before surgery. Subsequently, an intra-operative CT is acquired with the patient in the surgical position. The intra-operative CT is used to link the 3D anatomical model, based on preoperative data, with the actual OR setup, and to the reference sensor positions. During surgery, a tracked pointer is used, which is visible in the abdomen, and also virtually in the 3D anatomical model. Organs at risk that are relatively rigid with respect to the reference sensors, such as the iliac vessels and a large part of the ureters, can be located accurately using the pointer. This is also valid for pathologic lymph nodes which are located near the large vessels, as well as for tumours which are rigid due to invasion of pelvic structures. This system is being evaluated in an ongoing feasibility study (N13NAV, NL43553.031.13) for a wide range of indications. In this study, so far 37 patients have been included. We started with inclusion of only colorectal patients (n=21), but enthusiastic feedback and interest from other disciplines resulted in widening of the inclusion to patients with urologic (n=12), gynaecologic (n=1) and sarcoma tumours (n=3). In these procedures the navigation system was used to localize the borders of 4 primary tumours (all radical resections) and 6 local recurrences (5 radical resections). Furthermore, in 25 patients a total of 71 extra-regional LNs were identified on preoperative imaging. Of the 71 LNs, 67 were successfully retrieved using the developed navigation technique. Of the remaining 4 LNs, three in the obturator region were diminished to a size of less than 3 mm after neoadjuvant chemo-radiation. The fourth LN, originating from the prostate, was located within the mesorectum and was probably displaced during surgery. The different surgical disciplines adopted the navigation system rapidly, and provide patients to include in the study on a regular basis.

The running N13NAV is a feasibility study which will not provide any actual proof on superiority of surgical navigation over standard surgery. The promising results in the subgroup of patients with extra-regional lymph nodes have led to the design of the current prospective randomized controlled trial in which the beneficial value of the navigation system for abdominal extra-regional lymph node dissections will be evaluated.

2. OBJECTIVES

Primary Objective:

To evaluate if navigation assistance results in more successful localization and removal of extra-regional abdominal lymph nodes compared to standard surgery. Extra regional lymph nodes are defined as suspect target lymph nodes outside the standard resection area. Primary objective is a reduction in the number of failed procedure, in which failure is defined as: presence of any residual target lymph node on follow-up imaging.

Secondary Objective(s):

As secondary endpoints we will evaluate:

- Time from having the surgical field prepared for lymph node localization to actual removal of the lymph node
- Individual LN retrieval rate
- Overall surgery time
- Blood loss
- Operator satisfaction using standard questionnaires
- Grade ≥ 3 morbidity
- Cost
- Hospital stay
- Health related quality of life, using QLQ-C30 (all patients), CR29 (colorectal patients), PR25 (urology patients), and EQ-5D-5L for cost effectiveness

3. STUDY DESIGN

This study is designed as a prospective randomized controlled trial (Fig. 1). After giving informed consent, patients will be randomized between conventional surgery without navigation (control arm), and the experimental arm with navigation. Patients will be asked to answer health-related quality of life questionnaires before surgery, and at 3, 6 and 12 months after surgery. Included

patients are part of this study from the first trial related procedure, until 30 days after surgery, even though the questionnaires will take place up to 12 months after surgery. This is because navigation related complications will not take place after the first 30 days. All patients will undergo an additional baseline CT scan with intra-venous contrast in the 2 weeks before surgery. This scan will be used to assess the target LNs. In the experimental arm the CT scan will be used to create a 3D model of the pelvic anatomy including the target LNs.

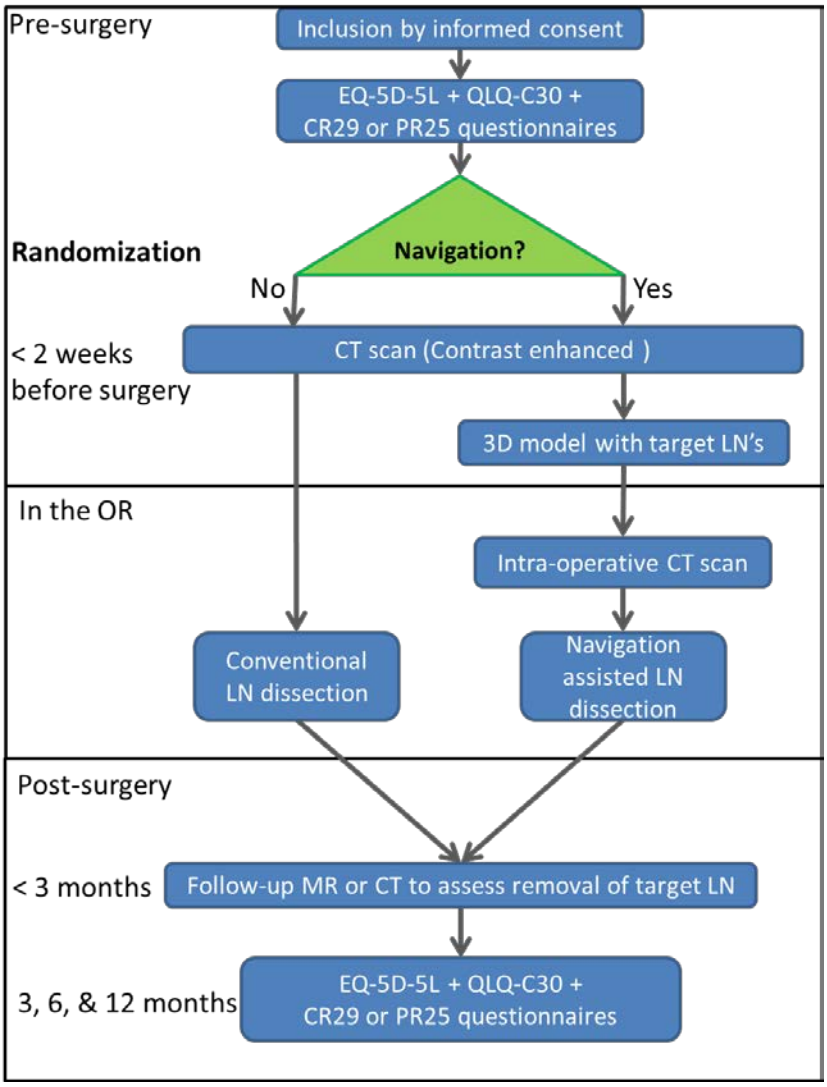

Fig. 1: outline of the study

We have chosen for a randomization setup to have minimum selection bias in the comparison between navigation and conventional surgery. Having a control arm is certainly valid, since the navigation setup is still experimental.

4. STUDY POPULATION

4.1 Population (base)

Eligible patients are patients over 18-years of age of the Netherlands Cancer Institute (NKI-AvL), who are scheduled for abdominal surgery by laparotomy. Removal of at least one extra-regional lymph node should be part of the surgical plan. Currently these patients are included in the navigation pilot study (N13NAV) at a rate of 1-2 patients per week. In the enrolment process the "patient informatie" will be used to inform the patients and the informed consent must be signed prior to the first trial related procedure.

4.2 Inclusion criteria

In order to be eligible to participate in this study, a subject must meet all of the following criteria:

- Age ≥ 18
- Scheduled for open abdominal surgery
- Planned removal of at least 1 extra-regional lymph node suspect on imaging, as assessed by the specialist who will perform the operation
- Informed consent

4.3 Exclusion criteria

A potential subject who meets any of the following criteria will be excluded from participation in this study:

- Metal implants in the pelvic area which could influence the 3D modelling or navigation accuracy
- Contra-indication for contrast enhanced CT scanning

4.4 Sample size calculation

In the current pilot study (N13NAV), a total of 71 targeted lymph nodes were identified in 25 patients. In total, we failed to localize 4 of the 71 lymph nodes, in four separate patients. We therefore expect to have approximately 16% (4/25) failed procedures in the navigation arm (p_B=0.16). In the current pilot study, surgeons indicated in at least 6 patients that certain localized lymph nodes would have not been found without navigation. We therefore estimate a failure rate of 40% (p_A=0.40) in the control arm ((6+4)/25). In our study setup we test the following hypotheses H_0: $p_A = p_B$ and the alternative hypothesis H_1: $p_A > p_B$ (one-sided alpha). The number of

needed patients per arm with an alpha of 0.05 and a power of 80% is 41. Accrual of all patients is estimated to take 36 months (excluding the time to the first follow-up scan for all).

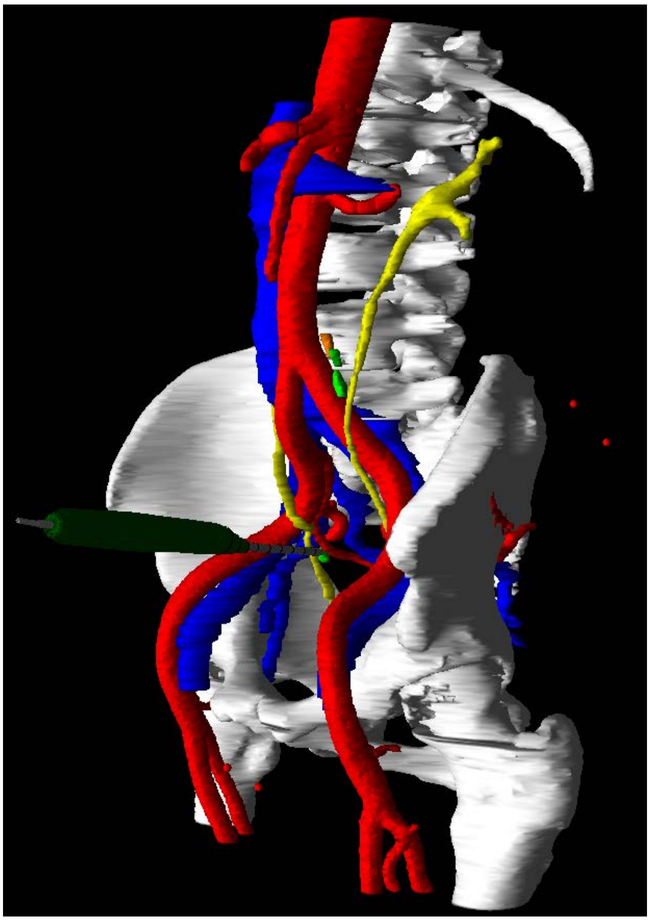

Fig. 2: Screenshot of the 3D anatomical model of a patient during navigation surgery in which the arteries (red), veins (blue), ureters (yellow), bones (white), and pathologic lymph nodes (lime) are shown. The tracked pointer is manipulated by the surgeon in the pelvis of the patient, and is shown in real-time within the 3D model (green). In this example a lymph node was localized in between the internal iliac artery, the internal iliac vein and the ureter

5. TREATMENT OF SUBJECTS

All included patients are already scheduled for surgery. Participation will not influence the waiting time. If patients decide to participate, additional burden is dependent on randomization. All patients will be asked to take quality of live and side effects questionnaires before surgery and at 3 and 12 months after surgery. The questionnaires take approximately 20 minutes per time point.

Patients who are randomized to the <u>conventional arm</u> will be operated according to current clinical practice. These patients will undergo one additional CT scan with contrast (10 minutes) in the two weeks before surgery on which the target lymph nodes will be indicated. In the control arm, no 3D anatomical models will be generated.

Patients in the <u>experimental arm</u> will be operated with assistance of the in-house developed navigation system. One extra contrast enhanced CT scan before surgery will be acquired in the two weeks before surgery (10 minutes). During surgery, three Patient Trackers are attached to the skin of the patient using medical tape, not obstructing the surgical field. An additional intra-operative CT scan is acquired (±10 minutes) for calibration, which will be an addition to the total time of

anaesthesia. The surgeon will have a sterile tracked navigation pointer which can be used to correlate the actual anatomy of the patient with a 3D model derived from pre-operative imaging (Fig. 2). With this setup, the surgeon is provided with a view beyond surgical plane. After opening the abdomen, some anatomical landmarks will be used to verify the navigation accuracy, which will take a maximum of 5 minutes. Subsequently, the navigation system can be used to localize the targeted lymph nodes.

We will try to plan the additional pre-operative CT scan on a day at which the patient is already scheduled to visit our hospital to limit the travel time. Logistical challenges might result in an additional hospital visit.

5.1 Investigational treatment

In this study patients will be randomized between conventional open surgery and open surgery with the assistance of the surgical navigation system. In the conventional approach, imaging findings are processed preoperatively, and translated in actions during surgery by the surgeons brain. With the navigation system the surgeon is provided with a direct real-time correlation between the pre-operative imaging findings and the actual patient anatomy. We hypothesize that the navigation assistance will result in more successful and faster localization of extra-regional lymph nodes. Follow-up imaging will be used to evaluate if all targeted LN are removed. If follow-up imaging with CT or MRI within 3 months after surgery is clinical routine, these data will be used. If imaging is not planned within 3 months, an additional CT scan will be planned by the treating physician.

5.2 Use of co-intervention (if applicable)

No specific restrictions for included patients are specified. Besides the study related procedures, included patients will follow the same pre-operative workup as non-included patients scheduled for a similar operation.

5.3 Escape medication (if applicable)

Not applicable

6. INVESTIGATIONAL PRODUCT

The navigation system used for this study is composed of elements of the Aurora Electromagnetic Measurement System from Northern Digital Inc. (Waterloo, Ontario, Canada) and is combined with in-house developed software for navigation and visualization. The system is developed for use in the NKI-AvL only, and there is no current intent to commercialize the system. According to Dutch national guidelines (beleidsplan medische apparatuur), and agreements between the METC and the department of clinical physics, a tailored safety management and risk assessment protocol is required. All safety management is recorded in the investigational medical device dossier (IMDD) used for all non-CE marked devices. This dossier is composed in close collaboration with the clinical physics department and is added to this application in supplement K1.

6.1 Name and description of investigational product(s)

An extensive description of the investigational product can be found in the IMDD. The navigation system is registered as RT NaviOne system in the NKI-AvL (ID0024519). The used hardware consists of an electromagnetic NDI Aurora V2 tracking system and a standard research PC with monitor mounted on a trolley with a galvanic isolated power supply. The Aurora system consists of an electromagnetic field generator, which is positioned between the table and the patient in a dedicated matrass, a system control unit on the trolley, and four sensor interface units which are mounted on the side of the bed. Three Philips Patient Trackers are used to track the position and orientation of the patient during surgery. One sterilized NDI Aurora straight tip probe is used by the surgeon to correlate the actual anatomy with the 3D model.

The used navigation software is in-house developed. An official software release is performed in collaboration with the clinical physics department (Michiel Sinaasappel) before clinical use. In the release process, the software will be tested on consistency and reliability, and documentation will be stored. The system will be tested in a black-box approach, where an entire chain-test from acquiring CT data until the actual navigation to known positions in a phantom is evaluated. In the navigation software, all available imaging information of the patient can be loaded, including 3D segmentations of the input scans which can be used as a 3D roadmap. In the program, user input is used to semi-automatically localize the Patient Trackers on imaging data. The actual positions of the Patient Trackers are continuously derived from the Aurora system, and the correlation between the 3D roadmap and the actual anatomy is automatically updated. Finally, the tracked probe is digitally projected in the 3D roadmap.

6.2 Summary of findings from non-clinical studies

The navigation system has been extensively tested in a pre-clinical setting (See also IMDD). The electromagnetic tracking accuracy can be influenced by surrounding electronics and ferromagnetic materials. Also, tracking accuracy is known to reduce with increasing distance to the field generator. We have evaluated the tracking accuracy of our navigation setup, and concluded that our OR setup has limited effect [14]. Furthermore, tracking accuracy was within 1 mm root-mean-square error (1SD) in the region between 12 cm (minimum distance) and 35 cm from the field generator. This is the actual region in which we can expect the Patient Trackers and the Probe, and is deemed accurate enough for the application.

6.3 Summary of findings from clinical studies

The navigation system is currently under investigation in a clinical pilot study in which we evaluate its feasibility and safety (N13NAV). The pilot study has been amended to include a maximum of 75 patients as a registration study. An interim analysis was performed after 37 patients. In terms of feasibility, only for one patient the navigation system could not be used during surgery, because the patient was not positioned correctly with respect to the field generator. In terms of safety, none of the procedures resulted in serious adverse events which could be contributed to the use of the navigation system. In all procedures the accuracy/correctness of the system is evaluated on obvious anatomical landmarks (pubic bone, iliac spine, promontory, bifurcations of aorta/iliac arteries) and is corrected if necessary. With the system we are able to localize internal anatomic structures with an accuracy <4 mm. In 4 patients, the navigation system was used for the localization of a locally advanced primary tumour, all resulting in R0 resections. In five out of six patients in which the system was used for localization of a local recurrence an R0 resection was achieved. In one gynaecological recurrence case no R0 was achieved, which was mainly due to a large setup difference between CT and the OR. This procedure was performed before we had intra-operative imaging, which would have been very beneficial. In 25 patients, a total of 71 target lymph nodes were identified on imaging. Sixty-seven out of 71 LNs were actually localized using the tracking system. The four missed LNs consisted of three obturator LNs which were diminished to a size of less than 3 mm after neoadjuvant treatment, and one mesorectal LN which was probably not rigid enough to be localized. Unfortunately, the latter patient developed a local recurrence at the position of the mesorectal LN. In total 15 different surgeons used the navigation system. All surgeons indicated improved anatomical insight, better orientation, and significant additional value of the navigation system. In our experience, use of the system is very intuitive, and novel users directly understand the principle.

6.4 Summary of known and potential risks and benefits

The main risk of the navigation setup is a miss-registration between the actual patient setup and the 3D anatomical model. If this happens, the surgeon can choose a dissection path through critical pelvic structures such as blood vessels, ureters or nerve tissue. To prevent these errors, obvious anatomical landmarks are used to evaluate the accuracy of the system.

Another potential risk is deformation of the patient anatomy. If this happens, the 3D anatomical model is not representative anymore for the actual anatomy. This risk is minimized by patient selection. The mobility of targets (lymph node, recurrence or primary tumour) is assessed before inclusion by evaluation of the location and position changes between different imaging sessions by imaging experts of the study (Bas Pouw & Jasper Nijkamp). Only targets which are expected to be rigid with respect to the surrounding bony anatomy or large vessels are included.

The major benefit of the navigation setup is improved anatomical insight during surgery. The navigation system provides a view beyond surgical planes. It provides insight in the optimal surgical approach, and aids in decision making between being radical and preventing morbidity. We have the experience that surgery is performed with more certainty and faster for the so far included cases, which were all deemed surgically challenging.

6.5 Description and justification of route of administration and dosage

Not applicable

6.6 Dosages, dosage modifications and method of administration

Not applicable

6.7 Preparation and labelling of Investigational Medicinal Product

Not applicable

6.8 Drug accountability

Not applicable

7. NON-INVESTIGATIONAL PRODUCT

There are no non-investigational products used in this study

8. METHODS

8.1 Study parameters/endpoints

8.1.1 Main study parameter/endpoint

The main study parameter is the percentage of failed procedures, in which failure is defined as: presence of any residual target lymph node on follow-up imaging.

On the pre-operative CT scans, target lymph nodes will be indicated and removal is planned. Cases where the surgeon intraoperatively assess inoperability of a LN due to expected morbidity will be recorded. The actual verification of removal will be done on the first follow-up scan. This should be a CT or MRI scan acquired within 3 months after surgery. The scan will first be registered to the pre-operative CT scan. Two observers (Bas Pouw & Jasper Nijkamp) will check for each target LN on the planning CT if they are not visible on the follow-up scan. In case of doubt, and for verification of image interpretation, a radiologist will be consulted.

8.1.2 Secondary study parameters/endpoints

Time to localization: to assess the time to localization, the surgeon will be asked to indicate when he/she is ready to start localizing a targeted LN. The start point is after opening the abdomen, clearance of adhesions, installation of an abdominal wall retraction device. Time will be recorded until the LN is localized, and also until the LN is removed.

Other parameters which will be recorded are: success rate of retrieved individual LNs, overall surgery time from start of surgery until the abdomen is closed again; blood loss; technical failures of the navigation system; and operator satisfaction using standard questionnaires. For health technology assessment, EQ-5D-5L, QLQ-C30, and QLQ-CR29 or QLQ-PR25 will be used before surgery, and at 3 and 12 months

8.1.3 Other study parameters (if applicable)

We will record body weight, length, age, gender, type of primary tumour, location of primary tumour, size of LN, neoadjuvant treatment, previous pelvic surgery, previous pelvic radiotherapy.

For the patients operated in the navigation arm, targeted LNs which are removed individually will each be evaluated by the pathologist. The pathological findings will be recorded, and correlated with pre-operative imaging parameters. This will provide more insight into prediction of the risk of LN involvement on imaging.

8.2 Randomisation, blinding and treatment allocation

Patients will be randomized according to a randomization scheme provided by the Trial bureau through a website. The randomization scheme is blinded from the surgeons and investigators. In the randomization we will stratify for surgical sub-specialization (surgery, urology, gynaecology), since differences in their approach to the targeted lymph nodes might exist. After receiving informed consent, the researcher will visit the randomization website, provide the specialism, and the patient will automatically be assigned a study number and treatment arm.

8.3 Study procedures

Preparation:

- All patients: a baseline CT scan with intravenous contrast will be acquired within 2 weeks before surgery, without bowel contrast. The contrast is used to highlight the arteries (first phase scan) and the ureters (late phase scan). The scan will contain the entire abdomen from the kidneys to the pelvic floor. The scan is acquired in routine clinical practice. (**10 minutes**)
- All patients: the baseline CT scan is registered with other available imaging data (MR/CT/PET), and a researcher will indicate the target lymph nodes. These targets are confirmed with the operating surgeon/urologist. If image interpretation is challenging, a radiologist will be consulted. (Responsibility of the **researcher**)
- Experimental arm: a 3D anatomical model will be generated by the researcher. The total model will contain the arteries, veins, ureters, bones, and if necessary nerves close to the target LNs. The total model will discussed with the surgeon before surgery. (Responsibility of the **researcher**)

In the OR:

- Experimental arm: in the OR the patient will be placed on a dedicated navigation table with a carbon fibre imaging part and the EM field generator will be integrated in the mattress. With the table it is possible to have straight, fixed leg supports, or adjustable

leg holders for French position in bowel surgery. Preparation of the OR table is responsibility of the **researcher**.

- Experimental arm: before surgery, two electromagnetic Patient Trackers will be placed on the back of the patient, left and right from the spinal column. A third Patient Tracker is positioned at the anterior side at the level of the pubic bone. (Responsibility of the **researcher**)
- Experimental arm: after the patient is anesthetised and positioned for surgery a 3D XperCT scan (no contrast fluid used) is acquired. (Responsibility of the **researcher**, **maximum 10 minutes**)
- Experimental arm: the 3D XperCT scan is registered to the planning CT based on bony anatomy. After registration, the locations of the Patient Trackers are determined. Subsequently, the navigation setup is standby for use. (Responsibility of the **researcher**)
- The trial arm of the patient is determined and surgery can start. (Responsibility of the **researcher**)
- Experimental arm: the accuracy of the navigation system is first checked by pointing at bony landmarks, such as the pubic bone, iliac spine, promontory, and/or the large vessels (e.g. the position of the aortic bifurcation or common iliac bifurcations). If needed, the navigation settings can be adapted in the software to improve accuracy. This process will take at most **5 minutes**. (Responsibility of the **surgeon and researcher**)
- All patients: During surgery, the following time points will be noted: start of surgery, surgical field ready for LN localization, LN localized, LN removed, end of surgery. (Responsibility of the **researcher**)
- Experimental arm: At the end of the surgery, the Patient Trackers are removed. (Responsibility of the **researcher**)
- Experimental arm: The surgeon/urologist is asked to fill out the satisfaction questionnaire. (Responsibility of the **researcher**)

After surgery:

- All patients: within 3 months after surgery, a follow-up scan should be acquired. In most cases a standard clinical follow-up scan is available, either a diagnostic CT scan, or an MR scan of the operated region. If not available, the patient will be scheduled for an extra diagnostic CT scan. (Organization is responsibility of the **researcher**, the **treating physician** is clinically responsible)

8.4 Withdrawal of individual subjects

Subjects can leave the study at any time for any reason if they wish to do so without any consequences. The investigator can decide to withdraw a subject from the study for urgent medical reasons.

8.4.1 Specific criteria for withdrawal (if applicable)

Not applicable

8.5 Replacement of individual subjects after withdrawal

Every subject that withdraws from the study will be replaced by a new one to adhere to the power calculations.

8.6 Follow-up of subjects withdrawn from treatment

There is no specific follow-up of the patients, except that the first follow-up scan is used to evaluate the primary endpoint. Therefore, study related follow-up of withdrawn patients is not performed.

8.7 Premature termination of the study

Reasons for premature termination of the study are:

- Occurrence of serious adverse events that are directly related to the navigation system.

9. SAFETY REPORTING

9.1 Temporary halt for reasons of subject safety

In accordance to section 10, subsection 4, of the WMO, the sponsor will suspend the study if there is sufficient ground that continuation of the study will jeopardise subject health or safety. The sponsor will notify the accredited METC without undue delay of a temporary halt including the reason for such an action. The study will be suspended pending a further positive decision by the accredited METC. The investigator will take care that all subjects are kept informed.

9.2 AEs, SAEs and SUSARs

9.2.1 Adverse events (AEs)

Adverse events are defined as any undesirable experience occurring to a subject during the study, whether or not considered related to the investigational product, trial procedure, or the experimental intervention. All adverse events reported spontaneously by the subject or observed by the investigator or his staff will be recorded.

9.2.2 Serious adverse events (SAEs)

All Serious Adverse Events (SAE) occurring from registration until 30 days after the last protocol treatment/administration should be reported through the web portal *ToetsingOnline* to the accredited METC that approved the protocol. Serious adverse events occurring more than 30 days after the last study medication/treatment will NOT be reported unless the investigator feels that the event may have been caused by the study treatment or a protocol procedure. Study-specific clinical outcomes of death because of disease progression are exempt from serious adverse event reporting, unless the investigator deems them related to use of the device.

A serious adverse event is any untoward medical occurrence or effect that

- results in death;
- is life threatening (at the time of the event);
- requires hospitalisation or prolongation of existing inpatients' hospitalisation;
- results in persistent or significant disability or incapacity;
- is a congenital anomaly or birth defect; or

- any other important medical event that did not result in any of the outcomes listed above due to medical or surgical intervention but could have been based upon appropriate judgement by the investigator.

An elective hospital admission will not be considered as a serious adverse event.

The investigator will report all SAEs to the sponsor without undue delay after obtaining knowledge of the events. The sponsor will report the SAEs through the web portal *ToetsingOnline* to the accredited METC that approved the protocol, within 7 days of first knowledge for SAEs that result in death or are life threatening followed by a period of maximum of 8 days to complete the initial preliminary report. All other SAEs will be reported within a period of maximum 15 days after the sponsor has first knowledge of the serious adverse events.

9.2.3 Suspected unexpected serious adverse reactions (SUSARs)

Not applicable since we don’t investigate a medicinal product

9.3 Annual safety report

Not applicable since we don’t have an investigational medicinal product. The investigational medical device has been evaluated on 37 patients in a previous study, without any safety issues.

9.4 Follow-up of adverse events

All AEs will be followed until they have abated, or until a stable situation has been reached. Depending on the event, follow up may require additional tests or medical procedures as indicated, and/or referral to the general physician or a medical specialist.

SAEs need to be reported till end of study within the Netherlands, as defined in the protocol

9.5 Data Safety Monitoring Board (DSMB) / Safety Committee

No data safety monitoring board or safety committee will be appointed.

10. STATISTICAL ANALYSIS

10.1 Primary study parameter(s)

The main study parameter is the percentage of failed procedures, where failure is defined as: presence of any residual target lymph node on follow-up imaging.

The final percentage of failed procedures in both arms will be compared using a one-sided two-proportion z-test. The evaluation will be done on intention to treat, including the lymph nodes which are marked as inoperable during surgery due to expected morbidity.

10.2 Secondary study parameter(s)

- Time from having the surgical field prepared for lymph node localization to actual removal of the lymph node: two-sample t-test,
- Overall surgery time: two-sample t-test
- The number of individual retrieved LN s in both arm, two-sample t-test
- Blood loss: two-sample t-test on all subjects
- Operator satisfaction: will be evaluated using standard questionnaires in the patients which are operated with navigation only. The questionnaire results in a score between 0 and 100. Technology which has a score 70 or higher is deemed beneficial [15].
- The QLQ and EQ-5D questionnaires are presented as a score from 0-100. Per arm these numbers will be handled as a continuous variable. We will also compare the change in the scores between T_0 and T_3 months, T_0 and T_6, and between T_0 and T_{12} months at a patient level per arm. A two-sample t-test will be used for statistical evaluation

10.3 Other study parameters

- body weight, length, age, gender, type of primary tumour, location of primary tumour, lymph node size, neoadjuvant treatment, previous pelvic surgery, previous pelvic radiotherapy will only be used to evaluate if there is a dis-balance between the arms.
- Correlation between pre-operative imaging findings and pathology: these data will only be used to generate new hypotheses

10.4 Interim analysis

There will be no interim analysis in this study.

11. ETHICAL CONSIDERATIONS

11.1 Regulation statement

The study will be conducted according to the principles of the Declaration of Helsinki and in accordance with the Medical Research Involving Human Subjects Act (WMO).

11.2 Recruitment and consent

Patients eligible for the study will be contacted during the pre-operative outpatient clinical appointment by the investigator, nurse practitioner, or treating physician. The investigators will contact the patient, after at least 3 days consideration, to ask if they want to participate. Informed consent will be obtained before the first trial related procedure.

11.3 Objection by minors or incapacitated subjects (if applicable)

Not applicable

11.4 Benefits and risks assessment, group relatedness

The use of a navigation system during surgery is mainly aimed at providing better anatomical insight during surgery. It is comparable to the use of navigation in daily traffic. There is a big chance that a destination can be reached without a navigation system, but with navigation the destination will be reached more often, faster, and along a shorter route without detours. The major risk of navigation is when a mismatch occurs between the actual anatomy and the anatomical model in the system. A mismatch can be caused by calibration errors, or by anatomical changes. To prevent calibration errors, the system is evaluated in every procedure using obvious anatomical landmarks. In the inclusion criteria we assess the possibility of having large anatomical changes of the location of the target lymph nodes.

Patients which will be operated in the conventional arm will get the standard of care in the Netherlands. Patients in the experimental arm will benefit for the estimated advantages of navigation. In the ongoing pilot study (N13NAV) we have the subjective experience that the use of navigation is beneficial in localization of targeted lymph nodes. The current study will bring objective evidence to this point.

11.5 Compensation for injury

The investigator has a liability insurance which is in accordance with article 7, subsection 6 of the WMO.

The sponsor has both a “General and Product Liability Insurance” and a “Clinical Trial Insurance”, which is in accordance with the legal requirements in the Netherlands (Article 7 WMO and the Measure regarding Compulsory Insurance for Clinical Research in Humans of 23rd June 2003).

These insurances provides cover for damage to research subjects through injury or death caused by the study.

1. € 450.000,-- (i.e. four hundred and fifty thousand Euro) for death or injury for each subject who participates in the Research;
2. € 3.500.000,-- (i.e. three million five hundred thousand Euro) for death or injury for all subjects who participate in the Research;
3. € 5.000.000,-- (i.e. five million Euro) for the total damage incurred by the organisation for all damage disclosed by scientific research for the Sponsor as 'verrichter' in the meaning of said Act in each year of insurance coverage.

The insurance applies to the damage that becomes apparent during the study or within 4 years after the end of the study.

11.6 Incentives (if applicable)

Not applicable

12. ADMINISTRATIVE ASPECTS, MONITORING AND PUBLICATION

12.1 Handling and storage of data and documents

Patient data will be handled confidentially and an identification code will be used to link the data to the subject. The principal investigator of the NKI safeguards the key to the code. In particular, for this study K.F.D. Kuhlmann, J. Nijkamp, Bas Pouw, Nikie Hoetjes, and T. Ruers will have access to the patient data. The handling of personal data complies with the Dutch Personal Data Protection Act (in Dutch: De Wet Persoonsbescherming). The signed informed consent statements, the listing of the identities of the patients, originals of the CRF's, and data collected during the study will be stored at the NKI.

12.2 Monitoring and Quality Assurance

Data will be recorded by a study coordinator (Nikie Hoetjes) using an electronic case report form. Every 6 months the quality and completeness of the data will be reported to the investigators.

12.3 Amendments

Amendments are changes made to the research after a favourable opinion by the accredited METC has been given. All amendments will be notified to the METC that gave a favourable opinion. Non-substantial amendments will not be notified to the accredited METC and the competent authority, but will be recorded and filed by the sponsor.

12.4 Annual progress report

The sponsor/investigator will submit a summary of the progress of the trial to the accredited METC once a year. Information will be provided on the date of inclusion of the first subject, numbers of subjects included and numbers of subjects that have completed the trial, serious adverse events/ serious adverse reactions, other problems, and amendments.

12.5 Temporary halt and (prematurely) end of study report

The investigator/sponsor will notify the accredited METC of the end of the study within a period of 8 weeks. The end of the study is defined as the last patient's last visit (follow-up scan).

The sponsor will notify the METC immediately of a temporary halt of the study, including the reason of such an action.

In case the study is ended prematurely, the sponsor will notify the accredited METC within 15 days, including the reasons for the premature termination.

Within one year after the end of the study, the investigator/sponsor will submit a final study report with the results of the study, including any publications/abstracts of the study, to the accredited METC.

12.6 Public disclosure and publication policy

Prior to initiation, the study will be submitted to the Netherlands National Trial register, which is a recognized and accepted by the World Health Organization and International Committee of Medical Journal Editors (ICMJE).

The study results will be submitted for publication in a peer-reviewed scientific journal.

13. STRUCTURED RISK ANALYSIS

In the IMDD we have described an extensive risk analysis on our investigative medical device.

14. REFERENCES

[1] E. Kapiteijn and C. J. H. van de Velde, "The role of total mesorectal excision in the management of rectal cancer.," *Surg. Clin. North Am.*, vol. 82, no. 5, pp. 995–1007, Oct. 2002.

[2] T. Pagliara, A. Nguyen, and B. Konety, "Contemporary lymphadenectomy templates: kidney, prostate and bladder cancer.," *Curr. Opin. Urol.*, vol. 24, no. 2, pp. 148–54, Mar. 2014.

[3] M. H. Albandar, M. S. Cho, S. U. Bae, and N. K. Kim, "Surgical management of extra-regional lymph node metastasis in colorectal cancer," *Expert Review of Anticancer Therapy*. Taylor & Francis, 07-Mar-2016.

[4] C. a. Jilg, H. C. Rischke, S. N. Reske, K. Henne, a. L. Grosu, W. Weber, V. Drendel, M. Schwardt, a. Jandausch, and W. Schultze-Seemann, "Salvage lymph node dissection with adjuvant radiotherapy for nodal recurrence of prostate cancer," *J. Urol.*, vol. 188, no. 6, pp. 2190–2197, 2012.

[5] P. L. Kubben, K. J. ter Meulen, O. E. M. G. Schijns, M. P. ter Laak-Poort, J. J. van Overbeeke, and H. van Santbrink, "Intraoperative MRI-guided resection of glioblastoma multiforme: a systematic review.," *Lancet Oncol.*, vol. 12, no. 11, pp. 1062–70, Oct. 2011.

[6] C. Senft, C. T. Ulrich, V. Seifert, and T. Gasser, "Intraoperative magnetic resonance imaging in the surgical treatment of cerebral metastases.," *J. Surg. Oncol.*, vol. 101, no. 5, pp. 436–41, Apr. 2010.

[7] J. Zwingmann, G. Konrad, E. Kotter, N. P. Südkamp, and M. Oberst, "Computer-navigated iliosacral screw insertion reduces malposition rate and radiation exposure.," *Clin. Orthop. Relat. Res.*, vol. 467, no. 7, pp. 1833–8, Jul. 2009.

[8] T. Y. C. So, Y.-L. Lam, and K.-L. Mak, "Computer-assisted navigation in bone tumor surgery: seamless workflow model and evolution of technique.," *Clin. Orthop. Relat. Res.*, vol. 468, no. 11, pp. 2985–91, Nov. 2010.

[9] K. Wu, N. P. Webber, R. A. Ward, K. B. Jones, and R. L. Randall, "Intraoperative navigation for minimally invasive resection of periarticular and pelvic tumors.," *Orthopedics*, vol. 34, no. 5, p. 372, May 2011.

[10] T. Langø, G. A. Tangen, R. Mårvik, B. Ystgaard, Y. Yavuz, J. H. Kaspersen, O. V Solberg, and T. A. N. Hernes, "Navigation in laparoscopy--prototype research platform for improved image-guided surgery.," *Minim. Invasive Ther. Allied Technol.*, vol. 17, no. 1, pp. 17–33, Jan. 2008.

[11] R. B. Bell and M. R. Markiewicz, "Computer-assisted planning, stereolithographic modeling, and intraoperative navigation for complex orbital reconstruction: a descriptive study in a preliminary cohort.," *J. Oral Maxillofac. Surg.*, vol. 67, no. 12, pp. 2559–70, Dec. 2009.

[12] A. Aschendorff, W. Maier, K. Jaekel, T. Wesarg, S. Arndt, R. Laszig, P. Voss, M. Metzger, and D. Schulze, "Radiologically assisted navigation in cochlear implantation for X-linked deafness

malformation.," *Cochlear Implants Int.*, vol. 10 Suppl 1, pp. 14–8, Jan. 2009.

[13] S. Atallah, B. Martin-Perez, and S. Larach, "Image-guided real-time navigation for transanal total mesorectal excision: a pilot study," *Tech. Coloproctol.*, vol. 19, no. 11, pp. 679–684, 2015.

[14] J. Nijkamp, B. Schermers, S. Schmitz, S. de Jonge, K. Kuhlmann, F. van der Heijden, J.-J. Sonke, and T. Ruers, "Comparing position and orientation accuracy of different electromagnetic sensors for tracking during interventions," *Int. J. Comput. Assist. Radiol. Surg.*, Jan. 2016.

[15] A. Bangor, P. T. Kortum, and J. T. Miller, "An Empirical Evaluation of the System Usability Scale," *Int. J. Hum. Comput. Interact.*, vol. 24, no. March 2015, pp. 574–594, 2008.
